# Season and size of urban particulate matter differentially affect cytotoxicity and human immune responses to *Mycobacterium tuberculosis*

**DOI:** 10.1371/journal.pone.0219122

**Published:** 2019-07-11

**Authors:** Srijata Sarkar, César E. Rivas-Santiago, Olufunmilola A. Ibironke, Claudia Carranza, Qingyu Meng, Álvaro Osornio-Vargas, Junfeng Zhang, Martha Torres, Judith C. Chow, John G. Watson, Pamela Ohman-Strickland, Stephan Schwander

**Affiliations:** 1 Department of Environmental and Occupational Health, Rutgers University School of Public Health, Piscataway, NJ, United States of America; 2 Department of Microbiology, Instituto Nacional de Enfermedades Respiratorias, México City, México; 3 Department of Pediatrics, University of Alberta, Edmonton, Alberta, Canada; 4 Duke Global Health Institute and Nicholas School of the Environment, Duke University, Durham, NC, United States of America; 5 Division of Atmospheric Sciences, Desert Research Institute, Reno, NV, United States of America; 6 Department of Biostatistics, Rutgers University School of Public Health, Piscataway, NJ, United States of America; 7 Department of Urban-Global Public Health, Rutgers University School of Public Health, Newark, NJ, United States of America; University of British Columbia, CANADA

## Abstract

Exposure to air pollution particulate matter (PM) and tuberculosis (TB) are two of the leading global public health challenges affecting low and middle income countries. An estimated 4.26 million premature deaths are attributable to household air pollution and an additional 4.1 million to outdoor air pollution annually. *Mycobacterium tuberculosis* (*M*.*tb*) infects a large proportion of the world’s population with the risk for TB development increasing during immunosuppressing conditions. There is strong evidence that such immunosuppressive conditions develop during household air pollution exposure, which increases rates of TB development. Exposure to urban air pollution has been shown to alter the outcome of TB therapy. Here we examined whether *in vitro* exposure to urban air pollution PM alters human immune responses to *M*.*tb*. PM_2.5_ and PM_10_ (aerodynamic diameters <2.5μm, <10μm) were collected monthly from rainy, cold-dry and warm-dry seasons in Iztapalapa, a highly populated TB-endemic municipality of Mexico City with elevated outdoor air pollution levels. We evaluated the effects of seasonality and size of PM on cytotoxicity and antimycobacterial host immunity in human peripheral blood mononuclear cells (PBMC) from interferon gamma (IFN-γ) release assay (IGRA)+ and IGRA- healthy study subjects. PM_10_ from cold-dry and warm-dry seasons induced the highest cytotoxicity in PBMC. With the exception of PM_2.5_ from the cold-dry season, pre-exposure to all seasonal PM reduced *M*.*tb* phagocytosis by PBMC. Furthermore, *M*.*tb*-induced IFN-γ production was suppressed in PM_2.5_ and PM_10_-pre-exposed PBMC from IGRA+ subjects. This observation coincides with the reduced expression of *M*.*tb*-induced T-bet, a transcription factor regulating IFN-γ expression in T cells. Pre-exposure to PM_10_ compared to PM_2.5_ led to greater loss of *M*.*tb* growth control. Exposure to PM_2.5_ and PM_10_ collected in different seasons differentially impairs *M*.*tb*-induced human host immunity, suggesting biological mechanisms underlying altered *M*.*tb* infection and TB treatment outcomes during air pollution exposures.

## Introduction

Two preventable conditions, *M*.*tb* infection and air pollution exposure, still cause enormous loss of human life worldwide. More than 10 million people develop tuberculosis (TB) and 1.4 million die from TB annually worldwide [1]. Urban outdoor air pollution in cities is one of the main environmental public health risk factors and estimated to have caused 4.2 million premature deaths globally in 2016 [2].

While most infections with *M*.*tb* remain asymptomatic, various factors contributing to immunosuppression increase the risk of TB development [3]. Exposure to tobacco smoke [4–8] and household air pollution [9–14] increase the risk of TB development. Recent studies also indicate that urban outdoor air pollution exposure increases the mortality of TB patients during TB therapy [15] and the risk of development of active TB [16, 17]. A potential link between long-term exposure to suspended particulate matter (PM) and increased rates of pulmonary TB has also been reported in North Carolina where levels of air pollution are relatively low [18].

Supporting the epidemiological evidence for adverse air pollution impacts on the natural course of *M*.*tb* infection, our group has demonstrated that diesel exhaust particles (DEP), a component of urban PM, impair human PBMC immune responses to *M*.*tb* via the downregulation of toll like receptor (TLR)-dependent cytokines (IFN-γ, IL-1β, IL-6, TNF-α) [19]. We have also shown that urban PM_2.5_ and PM_10_ induce cellular senescence and suppress *M*.*tb*-induced responses by impairing production of cytokines and antimicrobial peptides and growth control of *M*.*tb* in human respiratory epithelial cells [20]. In a recent study we have reported evidence that real-world inhalation-uptake of urban PM in human alveolar macrophages correlates with suppression of *M*.*tb* immunity in human lung and blood immune cells [21].

The size of PM during inhalation exposure is believed to determine affected target organs and health outcomes as PM_2.5_ penetrates terminal bronchioles and alveoli while the coarse fraction of PM_10_ primarily deposits in upper airways [22–24]. Differences in the physicochemical characteristics of urban PM resulting from seasonal differences in the content of agricultural and organic compounds, industrial production, vehicular traffic and home energy use may also modify interactions with biological systems [25, 26].

Seasonal variations of outdoor air pollution PM-related health effects have been demonstrated [27, 28] in epidemiological studies. Most biological studies, with the exception of a few [25, 29, 30], have been conducted with PM from selected seasons, thus evaluating health effects of PM with chemical composition characteristics representing short exposure periods only. However, physicochemical characteristics of urban PM could potentially be modified by seasonal effects and interactions with gaseous pollutants (NO_2_ and O_3_) that subsequently affect biological responses differentially [25, 26]. As the primary emissions and secondary formations of PM show seasonal variabilities, it is critical to consider seasonal variations of PM compositions while examining the health effects of PM exposures. Indeed, the toxic and inflammatory potential of PM has been shown to vary due to variations in chemical PM components based on differences in PM source locations and seasons [30–36]. PM size, season, composition and collection location have also been shown to affect PM-exposure-induced cardiovascular responses in rats [25].

In the current study, we examined *in vitro* the effects of exposures to urban air pollution PM from Iztapalapa, a municipality with a high prevalence of air pollution [37] and TB in Mexico City, on key components of protective human host immune responses to *M*.*tb*. PM_2.5_ and PM_10_ from rainy (R), cold-dry (CD) and warm-dry (WD) seasons, which vary in their chemical constitution, were examined regarding their capacity to induce cytotoxicity and modulate inflammatory PBMC responses to *M*.*tb*. Pre-exposures of PBMC to PM_2.5_ from R (R_2.5_), CD (CD_2.5_) and WD (WD_2.5_) and PM_10_ from R (R_10_), CD (CD_10_) and WD (WD_10_) seasons resulted in a dose-dependent inhibition of IFN-γ-production upon *M*.*tb* infection and stimulation with purified protein derivative (PPD), primarily in PBMC from IGRA+ subjects, i.e. persons with proven evidence of prior *M*.*tb* infection.

IL-1β-production was also inhibited, predominantly by CD_10_ and WD_10_ in *M*.*tb*-infected PBMC from both IGRA+ and IGRA- subjects. Further, upon PM pre-exposure, we observed a PM-size and season-dependent reduction of *M*.*tb* phagocytosis and *M*.*tb* growth control in monocyte-derived macrophages (MDM) and PBMC.

Combined, our findings suggest that exposure to urban outdoor air pollution PM of different size ranges and from different seasons modulate *M*.*tb*-induced immune responses, corroborating epidemiological findings of air pollution exposure effects on *M*.*tb* infection outcomes in the real-world.

## Materials and methods

Approval to perform this study, collect personal health information, and perform venipunctures was given by the Institutional Review Board (IRB) of Rutgers University, NJ (Protocol number: 2012001383) and the scientific and bioethics committee of the Instituto Nacional de Enfermedades Respiratorias Ismael Cosío Villegas in Mexico City, Mexico (INER, protocol B22-12). All study subjects provided signed written informed consent prior to any study interactions.

### Human subjects

Forty-two healthy subjects were recruited from staff, faculty and students at Rutgers and in Mexico City (the latter for Western Blot analysis only). Healthy nonsmoking men or women, 18–65 years of age, weighing at least 110 lbs., were included. Persons on anti-inflammatory medications were not eligible. At the initial study visit, 10 ml peripheral venous blood were collected and sent for T-SPOT.TB test (Oxford Diagnostic Laboratories, Memphis, Tennessee). This IGRA test determines the presence (IGRA+) or absence (IGRA-) of *M*.*tb* infection by quantifying frequencies of *M*.*tb* antigen-specific IFN-γ releasing blood T cells. During subsequent visits, approximately 100 ml of heparinized peripheral venous blood were collected to obtain PBMC for experimental studies.

### Collection of air pollution PM_2.5_ and PM_10_

PM_2.5_ and PM_10_ were collected from June 2012 to May of 2013 on the rooftop of the National Institute of Ecology and Climate Change (CENICA) in Iztapalapa, a highly populated, high vehicular traffic-exposed municipality of Mexico City, following the protocol described previously [20]. To collect adequate amounts of PM on modified nitrocellulose membranes, PM_2.5_ and PM_10_ was sampled for 24 h on Mondays, Wednesdays, and Fridays weekly using high-volume samplers (TE6070V-2.5, Tisch Environmental, Inc.; Village of Cleves, OH, USA, airflow rate 1.13 m^3^min^-1^) [38, 39]. The PM collection periods comprised the three seasons namely cold-dry (CD, November-February), warm-dry (WD, March-May) and rainy (R, June-October). Following careful mechanical recovery from the membranes, PM samples were pooled by particle size and months. Following measurements of the total monthly PM mass, PM samples were stored in baked glass vials at 4°C in desiccators in the dark. Seasonal bulk PM samples for *in vitro* studies and chemical analysis were subsequently generated by pooling monthly PM_2.5_ and PM_10_ samples according to their proportional contributions to the total mass of the total monthly PM samples included in the seasonal PM pools. PM samples were sterilized by autoclaving prior to use in *in vitro* exposure experiments.

### Analysis of PM chemical composition

PM_2.5_ or PM_10_ samples were combined for each size fraction by season and analyzed at the Desert Research Institute (Reno, NV) for 51 elements (from sodium to uranium) by X-ray fluorescence (XRF) [40]; eight ions (Cl^-^, NO_3_^-^, SO_4_^2-^ NH^+^, Na^+^, Mg^2+^, Ca^2+^, and K^+^) and levoglucosan by ion chromatography (IC) [41]; elemental carbon (EC) and organic carbon (OC) by thermal-optical analysis [41], and 114 polyaromatic hydrocarbons (PAH) by gas chromatography mass spectrometry (GC-MS) [42]. We analyzed non-autoclaved and autoclaved (at 121ºC, 30 minutes, 10.0 in. Hg in a SV1262 Prevac steam sterilizer, Steris, OH, USA) PM samples and no differences in the component composition were observed between them. Autoclaved PM was used in all experiments presented in the current study.

### Preparation of PM samples for *in vitro* use

Details of PM preparations for *in vitro* studies were described previously [20]. Briefly, R_2.5_, CD_2.5_, WD_2.5_, R_10_, CD_10_, and WD_10_ were weighed on a microbalance (CPA225D; Sartorius, Bohemia, NY, USA) and placed in prewashed and baked glass vials. Following sterilization by autoclaving at 121°C for 15 min, PM stock suspensions (1 mg/ml) were prepared in complete cell culture media phenol red free RPMI1640 (BioWhittaker, Lonza Walkersville, MD) supplemented with L-Glutamine (Thermo Fisher, Waltham, MA) and 10% human AB serum (Valley Biomedical, Inc., Winchester, VA)] and sonicated for 5 minutes at 100 Watts (351OR-DTH; Branson, Danbury, CT, USA). PM working suspensions (two-fold final concentrations) were prepared in 14 ml falcon tubes from stock suspensions, sonicated for 2 min and vortexed before adding onto cell cultures. All dilutions were prepared in complete cell culture media.

### Preparation of PBMC and MDM

PBMC and MDM preparations were performed as described previously [19, 43]. Briefly, heparinized peripheral venous blood was diluted with equal volumes of RPMI-1640. PBMC were obtained by density gradient centrifugation Ficoll-Paque (GE Healthcare Bio-Sciences AB, Sweden), counted in a hemocytometer and then resuspended at required concentrations in complete cell culture media for subsequent *in vitro* studies. Enriched CD14^+^CD3^-^ monocytes were generated by immunomagnetic separation from PBMC using a negative selection Pan Monocyte Isolation Kit (Miltenyi Biotec, Auburn, CA) according to manufacturer’s protocols. Monocytes (5 x 10^5^ per well) were then allowed to adhere on chamber glass slides (BD Falcon, Bedford, MA, USA). Non-adherent cells (lymphocytes) were removed by gentle washing. Adherent cells were cultured for differentiation to macrophages (MDM) at 37ºC in 5% CO_2_ in a humidified environment for seven days and then used for the *M*.*tb* phagocytosis assay.

### Preparation of *M*.*tb*

*M*.*tb* H37Ra (ATCC 25177, Manassas, VA) was grown in Middlebrook 7H9 broth medium (Sigma-Aldrich, Fluka. MO) and *M*.*tb* stock harvested following 21 days of incubation at 37°C in a shaking incubator. *M*.*tb* cultures were then centrifuged at 2095 x *g* (3000 rpm) for 30 min at room temperature (RT), suspended in Middlebrook 7H9 broth medium with 6% glycerol (Fisher Scientific, Fair Lawn, NJ), aliquoted in cryotubes and stored at –86°C until use. Concentrations of the *M*.*tb* stock were assessed by counting colony-forming units (CFU) from serial dilutions on 7H10 agar plates (Sigma-Aldrich, Fluka. MO). For the infection of PBMC or MDM, *M*.*tb* aliquots were thawed, centrifuged at 6000 x *g* for 5 min, suspended in 1 ml complete culture medium and de-clumped by vortexing with five 3 mm sterile glass beads followed by centrifugation at 350 x *g* for 2 min. Supernatants containing single *M*.*tb* suspensions were used for *in vitro* infections of PBMC or MDM.

### Lactate dehydrogenase (LDH) assay

To assess cellular viability upon PM exposure, freshly isolated PBMC (10^5^/well) were exposed to 0.1, 1 and 10 μg/ml of PM suspensions or left unexposed for 20 h in 96-well tissue culture plates. PM-exposed PBMC were also subsequently infected with *M*.*tb* at MOI1 (multiplicity of infection 1, infection with 1 bacterium/monocyte) or left uninfected. Following a 20 h incubation (37ºC in 5% CO_2_), cell supernatants were collected and LDH leakage (a marker of cytotoxicity) assayed (Promega, Madison, WI) as described previously [20, 43]. Briefly, 50 μl cell culture supernatants were incubated with 50 μl of substrate (CytoTox 96 Non-radioactive cytotoxicity Assay, Promega, Madison, WI) in a 96-well assay plate at room temperature for 30 minutes in the dark. Stop solution (50 μL) was added to each well and absorbance determined at 493 nm. Cellular toxicity was defined as percent (%) LDH leakage from cells calculated from the ratios of ODs of PM-exposed PBMC (after background subtraction) to the ODs of unexposed PBMC (after background subtraction) x 100. Supernatants from unexposed PBMC and SDS lysed PBMC served as negative and positive controls, respectively. OD of cell culture mediun served as background.

### ELISPOT assays

Frequencies of IFN-γ and IL-1β-producing PBMC upon PM exposure and *M*.*tb* infection were assessed by ELISPOT assays, as described previously [19]. Briefly, freshly isolated PBMC (2 x 10^5^/well for IFN-γ and 10^5^/well for IL-1β) were exposed to 0 (No PM), 0.1, 1 and 10 μg/ml of PM in triplicate in 200 μl complete cell culture media in high protein-binding 96-well plates coated with appropriate amounts of either IFN-γ or IL-1β antibodies.

Following incubation at 37ºC in 5% CO_2_ in a humidified environment for 20 h, PBMC were infected with *M*.*tb* at MOI1 or exposed in complete culture media to PPD. After incubations, PBMC were removed by washing, and ELISPOT plates developed according to manufacturer’s protocols. Plates were then scanned for image analyses of IFN-γ and IL-1β-producing cells (spot frequencies) with an automated ELISPOT reader (Immunospot series 5 analyzer, software version 5.0; Cellular Technology, Cleveland, OH). Frequencies of cytokine-producing cells were calculated by averaging spot numbers from triplicate wells for each experimental condition and plotted as functions of PM type, PM concentration and seasonal PM source.

### *M*.*tb* phagocytosis assay

MDM were pre-exposed in 8-well chamber glass slides (Thermo Nunc Lab-Tek II. NJ. USA) to R_2.5_, CD_2.5_, WD_2.5_, R_10_, CD_10_ or WD_10_ at final concentrations of 1 and 5 μg/ml and incubated at 37°C for 18 h. Pilot experiments using 10 μg/ml of PM_2.5_ and PM_10_ had shown too many particle inclusions in MDM making accurate counts of phagocytosed *M*.*tb* impossible.

Following PM pre-exposure, MDM were washed three times with RPMI1640 to remove non-phagocytosed PM, infected with *Mtb* at MOI 1 for 2h (37ºC, 5% CO_2_), and again washed three times with RPMI1640 to remove extracellular mycobacteria. Chamber glass slides were fixed with methanol (Bio Whittaker, Basel, Switzerland, stained with Kinyoun Staining Kit (AlphaTec. WA. USA), covered with coverslips (Sigma Aldrich, Saint Louis, MO) using slide mounting fluid (Fisher Scientific, Waltham, MA). Numbers of acid-fast bacilli in MDM were counted by bright field microscopy (Zeiss Axio Lab.A1 Laboratory Microscope, Göttingen Germany, 1,000x, oil immersion). Proportions of MDM with intracellular *M*.*tb* were determined within a total of 300 randomly selected MDM for each experimental condition.

### *M*.*tb* growth control assay

PBMC (2 x 10^5^) from IGRA- and IGRA+ subjects were exposed to R_2.5_, CD_2.5_, WD_2.5_, R_10_, CD_10_ or WD_10_ at final concentrations of 0, 1, and 5 μg/ml in 96-well round bottom tissue culture dishes (Corning Incorporated, Corning, NY) and incubated at 37°C for 18 h followed by centrifugation at 1200 rpm (335 x *g*) for 5 minutes. Supernatants containing non-phagocytosed PM were then carefully removed, PBMC washed twice and infected with *M*.*tb* at MOI1. Following 2-h infections, plates were washed three times to remove non-phagocytosed *M*.*tb* and plates incubated in complete culture media. On days 0 (1 h post infection), 1, 4 and 7, PBMC were lysed using 0.1% SDS for 10 min at room temperature followed by neutralization with 20% bovine serum albumin in Middlebrook 7H9 broth. Serial dilutions of cell lysates were then plated in triplicate on 7H10 agar plates and incubated at 37°C for 21 days when colony forming units (cfu) were assessed as described [20]. Observations of PM effects on *M*.*tb* growth control show day 4 data only.

### Western blotting to assess T-bet expression levels

To identify the expression levels of T-box transcription factor (T-bet), PBMC were exposed to 0, 1 and 10 μg/ml of PM_2.5_ annual bulk used in Mexico City for human bronchoalveolar cell exposures [21] in culture media for 24 h followed by *M*.*tb* infection at MOI1 or MOI5 or left uninfected for an additional 24h.

Following incubation, PBMC were lysed with RIPA (radio immunoprecipitation assay) lysis buffer system (Santa Cruz Biotechnology, Dallas, TX). Protein lysates were analyzed by SDS/PAGE followed by transfer onto polyvinylidene difluoride (PVDF) membranes. T-bet and GAPDH-specific proteins were analyzed by western blotting with specific antibodies (Cell Signaling Technology, Danvers, MA).

### Statistical analysis

Means and standard deviations summarized frequencies of cytokine-producing PBMC for PM size, PM dose, PM season, infections (*M*.*tb*), stimulant (PPD) and IGRA status. To examine the effects of PM size fractions, PM doses, PM seasons, and stimulants on frequencies of cytokine-producing PBMC, we used mixed linear regression models. Random intercepts for subjects accounted for correlation between measurements of samples from the same individuals. We considered each factor (PM size, dose, season, and stimulant) separately, and stratified by levels of all other factors. In addition, analyses were conducted for the combined group of individuals as well as stratified by IGRA status (positive +/negative-). F-tests were used to assess significance overall, while t-tests were used to examine pairwise differences. Additional tests examined whether IGRA status modified the effect of each factor through adding the cross product between the indicators for IGRA status and the factor in addition to main effects to the regression model and conducting an F-test of the interaction term. Sensitivity analyses using log-transformed counts rather than the raw counts as the response, given the somewhat skewed nature of some of the counts, yielded similar results.

Statistical comparisons of season and size differences in inorganic and organic components found in samples collected over a 12-month period representing three seasons were performed as follows. Mixed linear models controlling the Family-wise error rate at 0.05 was used to identify components that showed predictable variation. In particular, differences between component concentrations in (1) particle size fractions (PM_2.5_ vs. PM_10_) controlling for month and particle size, (2) rainy vs. cold-dry vs. warm-dry within PM_2.5_ and PM_10_ samples and (3) autoclaved versus non-autoclaved controlling for month (data not shown), were examined. In the first set of analyses, month and size were controlled as random effects. In the third case, only months in which the samples were both autoclaved and not autoclaved were included. For each set of analyses, *p*-values were calculated for each component and examined via QQ-plots. Benjamini-Hochberg’s procedure was used to control for multiple comparisons.

## Results

### Characterization of PM_2.5_ and PM_10_ samples from different seasons

To examine whether PM of different size and seasons vary in composition, we analyzed the main chemical components of the seasonal PM_2.5_ and PM_10_. The concentrations (ng/mg) of inorganic and organic components showing significant differences between PM_2.5_ and PM_10_ are presented in (S1 Table). Higher concentrations of organic compounds were observed in PM_2.5_ compared to PM_10_. Higher Ca concentrations were observed in PM_10_ than in PM_2.5_ samples, while V, Zn, Pb contents were higher in PM_2.5_ than PM_10_ samples. Anthropogenic emission products thus seem to be higher in concentration in PM_2.5_, while elements from the soil are present in higher concentrations in PM_10_.

Only a few statistically significant differences in the concentrations of the chemical components of PM_2.5_ and PM_10_ were noted comparing the PM from the three seasons. CD_10_ showed higher levels of ba-hopane (C30ba-hopane), benzo[e]pyrene, benzo[j+k]fluoranthene, benzo(ghi)fluoranthene, and benzo[a]pyrene contents than R_10_ or WD_10_ (S2 Table). R_2.5_ showed higher ba-hopane (C30ba -hopane) content than CD_2.5_ or WD_2.5_; and CD_2.5_ showed higher benzo[e]pyrene content compared to R_2.5_ or WD_2.5_ (S3 Table). Endotoxin contents in monthly PM samples were very low (S4 Table).

### Cytotoxicity of PM_2.5_ and PM_10_ from different seasons

**Fig 1:**
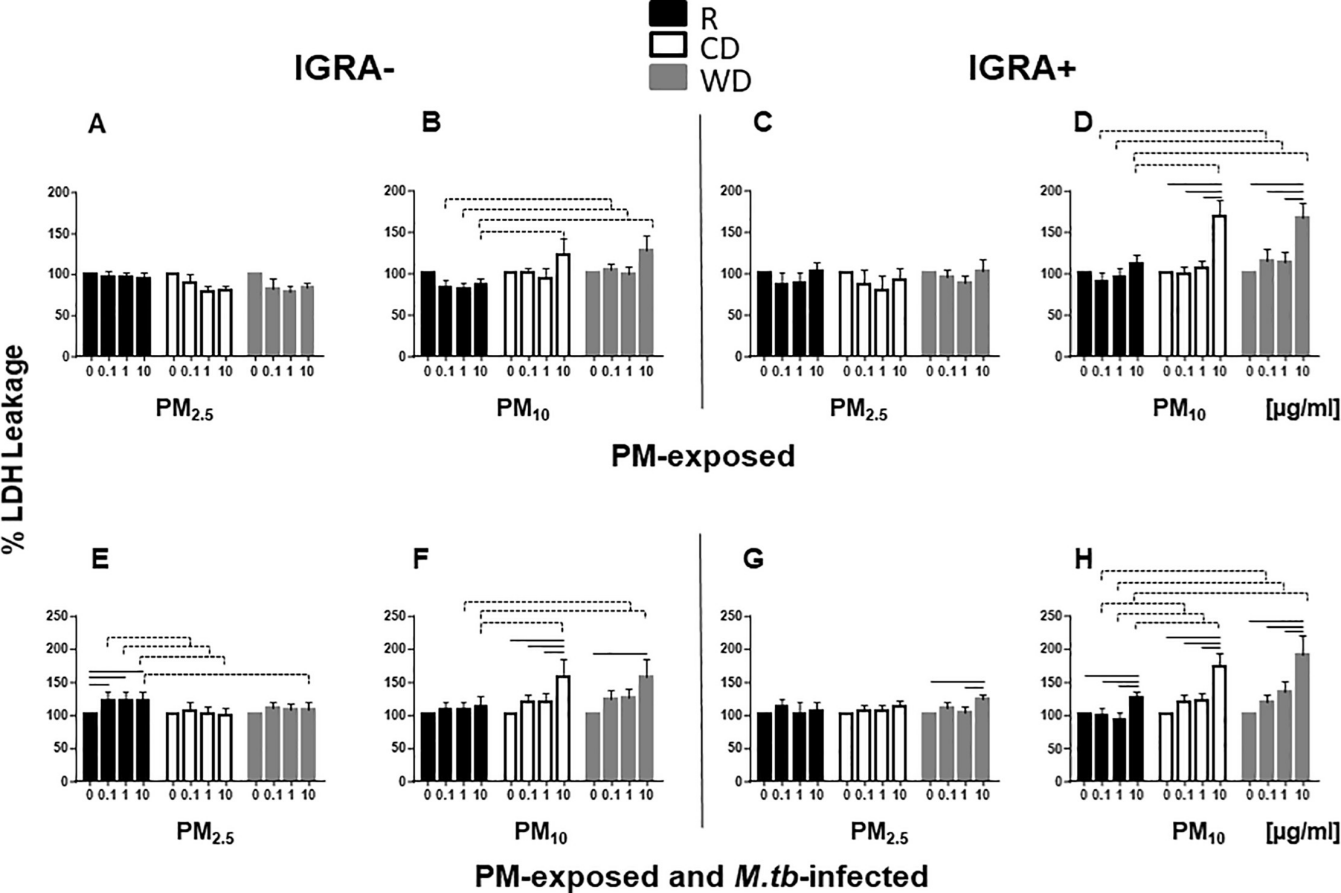
PM-induced cytotoxicity. PBMC from IGRA- (n = 6) and IGRA+ (n = 6) subjects were exposed in cell culture to 0 (no PM), 0.1, 1 and 10 μg/ml of R_2.5_, CD_2.5_, WD_2.5_, R_10_, CD_10_, WD_10_ at 37ºC, 5% CO_2_ in a humidified environment for 20 h. Following exposure to PM, PBMC were infected with *M*.*tb* at MOI1 (**E, F, G, H**) or left uninfected (**A, B, C, D**) and incubated for an additional 20 h. Culture supernatants were analyzed by LDH assay as described in Materials and Methods. Cytotoxicity is expressed as % LDH leakage (OD of treated—media background / O.D. of control—media background) x100. Results are shown as means ± SEM and statistical significances between doses (*p* ≤0.5) relative to unexposed PBMC (no PM) in **A-D** and *M*.*tb*-infected PBMC in **E-H** are shown with solid lines. Statistically significant differences between seasons are shown with dotted lines.

To assess the effect of exposure to PM from different seasons and each size fraction on the cell viability, PBMC were exposed to 0, 0.1, 1 and 10 μg/ml of R_2.5_, CD_2.5_, WD_2.5_, R_10_, CD_10_ or WD_10_ for 20 h followed by infection with *M*.*tb* MOI1 or left uninfected for an additional 20h, and LDH leakage, as a measure of cytotoxicity, assessed. No significant increases in LDH leakage were observed in PBMC from either IGRA- (Fig 1A) or IGRA+ (Fig 1C) subjects after exposure to R_2.5_, CD_2.5_ or WD_2.5_. Similarly, exposure to R_10_, CD_10_ or WD_10_ did not significantly increase LDH leakage in PBMC from IGRA-subjects (Fig 1B). LDH leakage was, however, increased significantly in PBMC from IGRA+ subjects exposed to 10 μg/ml of CD_10_ or WD_10_ but not R_10_ (Fig 1D).

Pre-exposure to R_2.5_ (Fig 1E) and CD_10_ and WD_10_ (Fig 1F) caused more cytotoxicity in *M*.*tb*-infected PBMC from IGRA- subjects than in *M*.*tb*-infected, but PM-unexposed PBMC. In IGRA+ subjects, pre-exposure to 10 μg/ml WD_2.5_ (Fig 1G) as well as R_10_, CD_10_ and WD_10_ (Fig 1H) followed by *M*.*tb* infection resulted in statistically significant increases in cytotoxicity relative to *M*.*tb*-infected but PM-unexposed PBMC. The reasons for the decreases in LDH levels relative to unexposed control PBMC, noted in certain conditions, are not clear.

Seasonal and size variations in PM-induced cytotoxicity were observed. CD_10_ (10 μg/ml) and WD_10_ (1 and 10 μg/ml) were significantly more cytotoxic than R_10_ (Fig 1B, 1D, 1F and 1H), irrespective of the IGRA status of the study subjects. CD_10_ and WD_10_ (Fig 1B, 1D, 1F and 1H) induced more cytotoxicity than CD_2.5_ and WD_2.5_ (Fig 1E and 1G), suggesting a possible PM size-dependent difference in cytotoxicity, particularly at the higher exposure concentration (Table 1).

**Table 1:** Effect of PM size on viability of PM-exposed *M*.*tb*-infected PBMC.

	R10 vs. R_2.5_			CD_10_ vs. CD_2.5_			WD_10_ vs. WD_2.5_		
	0.1 μg/ml	1 μg/ml	10 μg/ml	0.1 μg/ml	1 μg/ml	10 μg/ml	0.1 μg/ml	1 μg/ml	10 μg/ml
**IGRA-**	n.s	n.s	n.s	n.s	n.s	**CD**_**10**_**>CD**_**2.5**_**, *p* = 0.036**	n.s	n.s	**WD**_**10**_**>WD**_**2.5**_ ***p* = 0.038**
**IGRA+**	n.s	n.s	n.s	n.s	n.s	**CD**_**10**_**>CD**_**2.5**_ ***p* = 0.004**	n.s	n.s	**WD**_**10**_**>WD**_**2.5**_ ***p* = 0.042**

n.s. not statistically significant

In summary, these data provide evidence that urban PM-induced cytotoxicity predominantly relates to PM dose, size and season, suggesting that the composition of PM_10_ from CD and WD seasons may be responsible for the differences noted in comparison with PM_10_ from R season.

### Effect of PM exposure on *M*.*tb* phagocytosis in MDM

**Fig 2:**
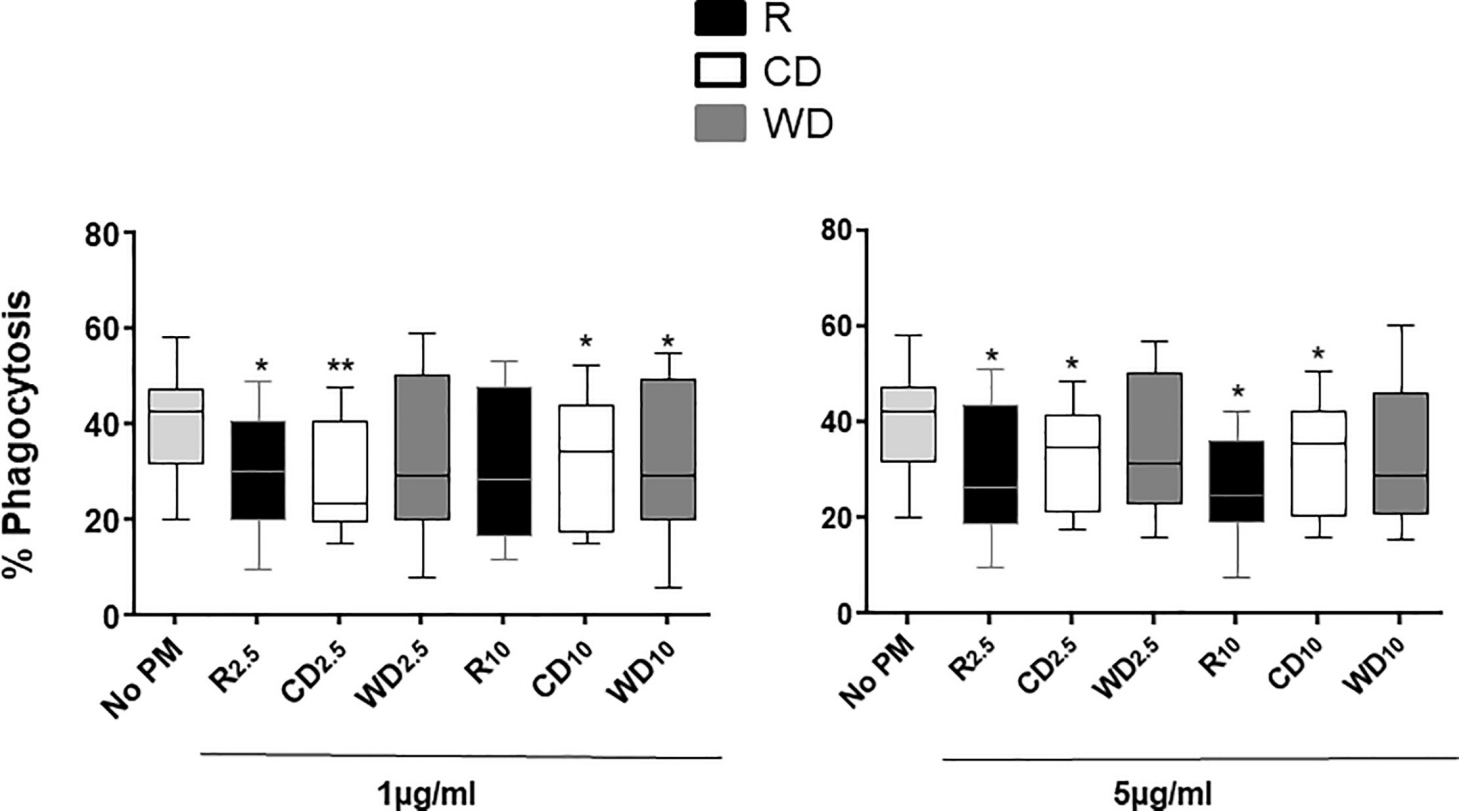
PM effects on *M*.*tb* phagocytosis. MDM were exposed to 1 and 5 μg/ml of R_2.5_ (n = 10, 1 μg/ml; n = 8, 5 μg/ml), CD_2.5_ (n = 9), WD_2.5_ (n = 9), R_10_ (n = 9, 1 μg/ml; n = 7, 5 μg/ml), CD_10_ (n = 9), and WD_10_ (n = 9) or left unexposed (n = 11) at 37ºC, 5% CO_2_ in a humidified environment for 18 h. Following exposure to PM, MDM were infected with *M*.*tb* at MOI1 for 2 h. Phagocytosis of *M*.*tb* by MDM was assessed by identification of acid-fast bacilli (Materials and methods). Proportions of MDM with intracellular *M*.*tb* were determined by bright field microscopy (1000x, oil immersion) within a total of 300 MDM in each experimental condition. Data are presented as medians, interquartile ranges (IQR) and 5^th^ and 95^th^ percentiles. Statistical comparisons were done by non-parametric Wilcoxon matched pair signed rank test. Statistically significant changes relative to *M*.*tb*-infected (no PM control) PBMC within each dose are shown with * (*p* ≤0.05) or ** (*p* ≤0.001).

To assess the effect of PM exposure on *M*.*tb* phagocytosis, MDM were exposed to 0, 1 or 5 μg/ml of PM_2.5_ and PM_10_ from the three seasons. PM pre-exposed MDM were then infected with *M*.*tb* at MOI1. *M*.*tb* phagocytosis was significantly reduced in MDM following pre-exposure to 1 and 5 μg/ml of R_2.5_ and CD_2.5_ (but not WD_2.5_) compared to unexposed MDM. Pre-exposure to R_10_ (5 μg/ml), CD_10_ (1, 5 μg/ml) and WD_10_ (1 μg/ml) followed by *M*.*tb* infection significantly reduced phagocytosis of *M*.*tb* compared to unexposed (No PM) *M*.*tb*-infected MDM (Fig 2). The effect of PM collection season on *M*.*tb* phagocytosis was greater than that of PM size.

### PM-induced IFN-γ and IL-1β production

**Fig 3:**
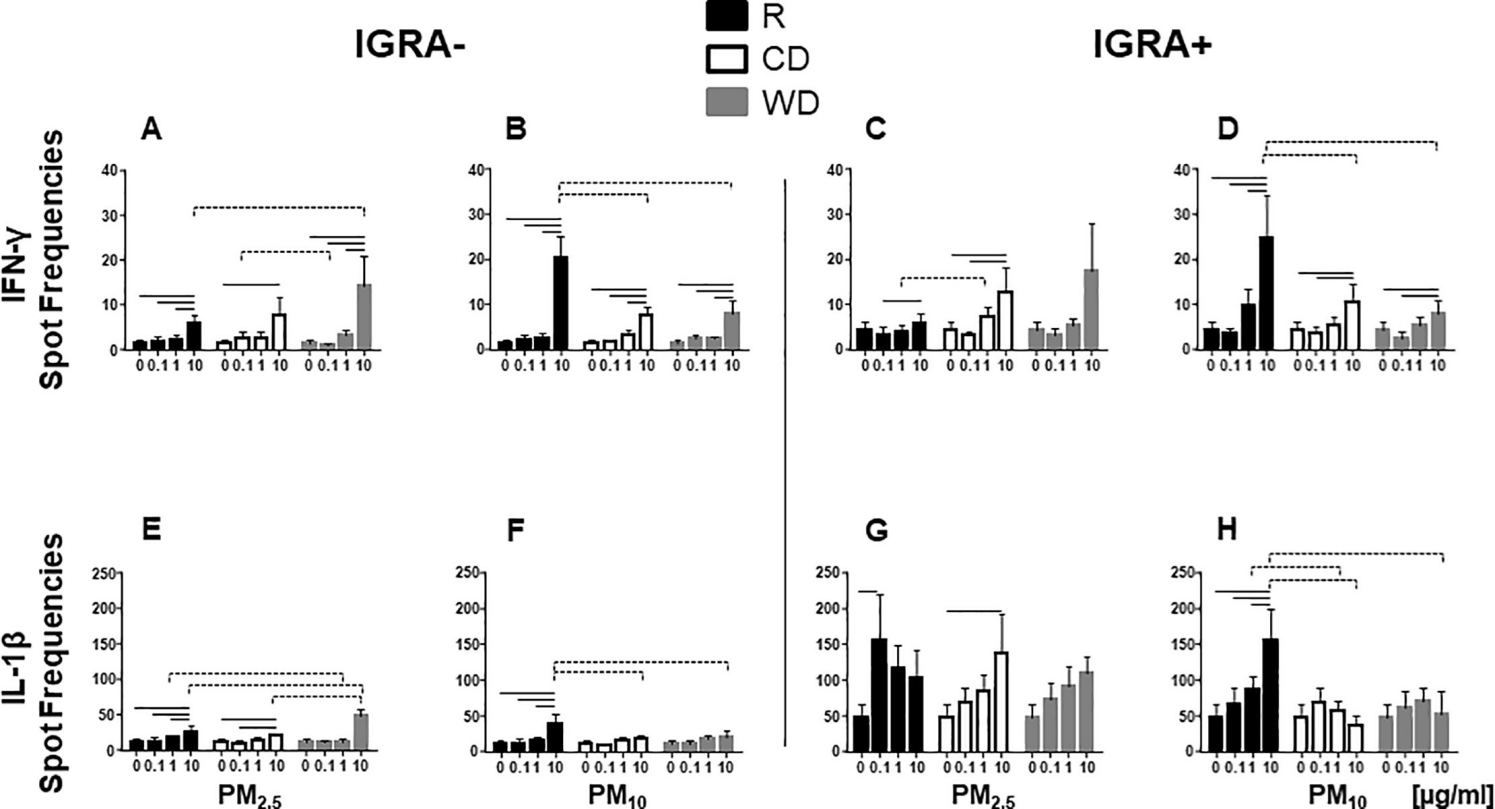
PM-induced IFN-γ and IL-1β production. PBMC from IGRA- (n = 8 for IFN-γ and n = 7 for IL-1β) or IGRA+ (n = 7) subjects were exposed to 0 (unexposed control), 0.1, 1 and 10 μg/ml of R_2.5_, CD_2.5_, WD_2.5_, R_10_, CD_10_, or WD_10_ or left unexposed at 37ºC, 5% CO_2_ in a humidified environment for 40 h. Frequencies of IFN-γ and IL-1β-producing PBMC were examined by ELISPOT assays (see Materials and Methods). Results are shown as mean ± SEM. Statistically significant differences (*p*≤0.05) between doses relative to unexposed control PBMC are shown with solid lines. Statistically significant differences between seasons are shown with dotted lines.

IFN-γ and IL-1β are critical components of the protective human host immune response to *M*.*tb* and predominantly produced by T-cells and monocytes, respectively. To explore whether exposure to PM of different sizes and seasons induces proinflammatory responses in PBMC, frequencies of IFN-γ and IL-1β-producing PBMC were examined by ELISPOT assays (Fig 3) following PM exposure. PBMC from IGRA- and IGRA+ subjects were exposed to 0, 0.1, 1 and 10 μg/ml of R_2.5_, CD_2.5_, WD_2.5_, R_10_, CD_10_ or WD_10_.

Frequencies of IFN-γ-producing cells increased significantly, irrespective of PM size and PM season, at 10 μg/ml concentrations in PBMC from both IGRA- and IGRA+ subjects, with the exception of WD_2.5_ exposure in PBMC from IGRA+ subjects (Fig 3A–3D). The higher frequencies of IFN-γ-producing PBMC upon exposure to R_10_ 10 μg/ml in both IGRA- and IGRA+ subjects compared to the frequencies induced by CD_10_ or WD_10_ (Fig 3B–3D) may be attributable to the lower cytotoxicity noted with R_10_ compared to that of CD_10_ or WD_10_ (at 10 μg/ml) (Fig 1B–1D).

Frequencies of IL-1β-producing PBMC increased significantly upon exposures to R_2.5_, CD_2.5_ and R_10_, particularly at 10 μg/ml, in PBMC from both IGRA- and IGRA+ subjects (Fig 3E–3G). Intriguingly, higher frequencies of IL-β-producing PBMC were observed in IGRA+ than in IGRA- subjects (compare Fig 3E and 3F with 3G and 3H).

Unlike the observed dose-dependent increase in the frequencies of IFN-γ-producing PBMC upon exposure to CD_10_ or WD_10_ (Fig 3B–3D), no such significant increases were observed assessing the frequencies of IL-1β-producing PBMC (Fig 3F–3H). As IFN-γ and IL-1β are predominantly products of T-cells and monocytes, respectively, these results suggest that exposures to PM from different seasons impact PBMC subpopulations differentially.

### PM effects on *M*.*tb* and PPD-induced IFN-γ and IL-1β production

We further investigated whether PM pre-exposure modifies host immune responses to *M*.*tb* and PPD. PPD is a gemisch of culture filtrate-derived *M*.*tb* proteins used in tuberculin skin testing. PBMC were exposed to R_2.5_, CD_2.5_, WD_2.5_, R_10_, CD_10_ or WD_10_ followed by infection with *M*.*tb* MOI1 or stimulation with PPD.

**Fig 4:**
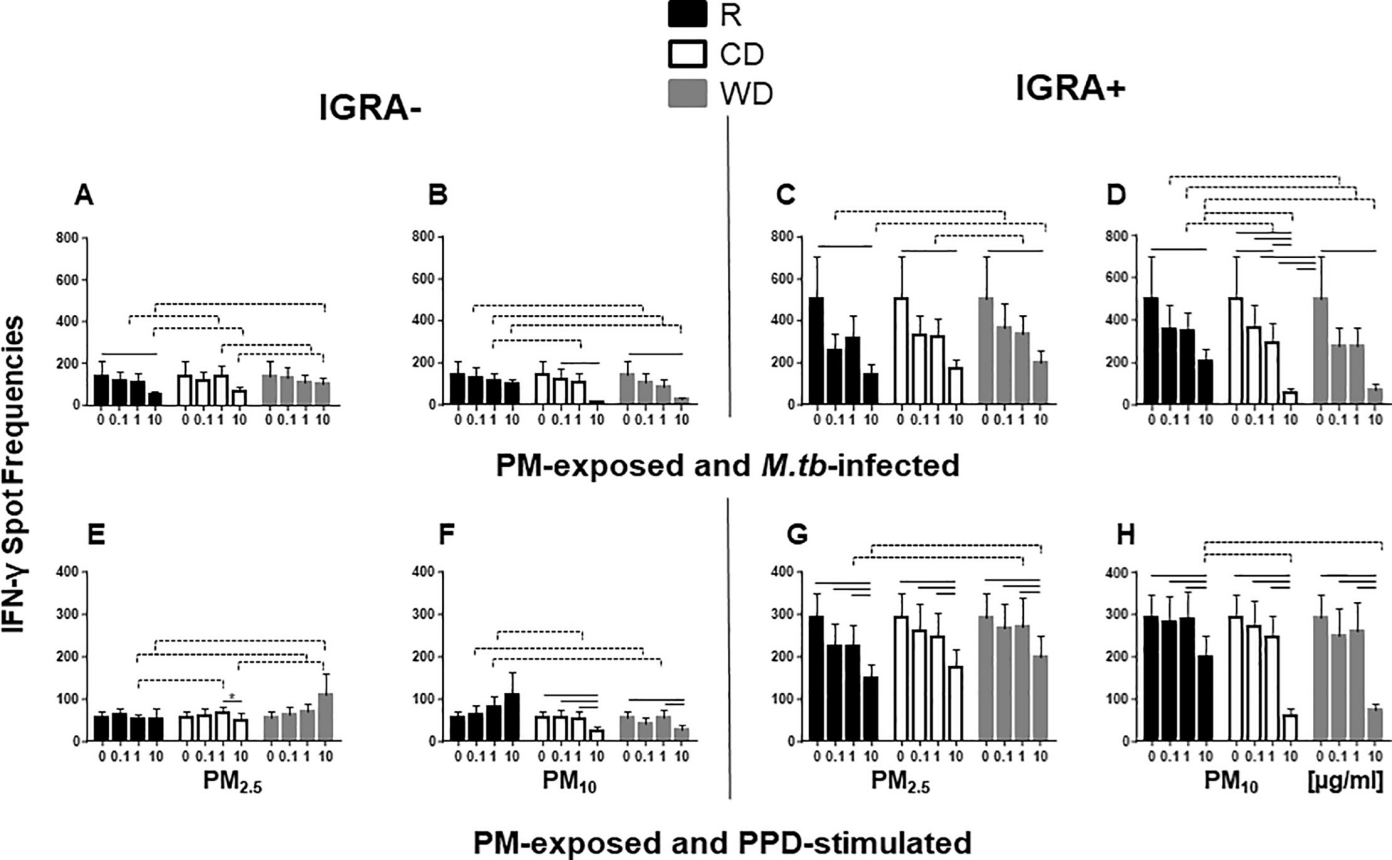
Effect of PM on *M*.*tb* and PPD-induced IFN-γ production. PBMC from IGRA- (n = 8 for *M*.*tb* and n = 6 for PPD) or IGRA+ (n = 7 for *M*.*tb*, n = 7 for PPD) subjects were exposed to 0 (no PM), 0.1, 1 and 10 μg/ml of R_2.5_, CD_2.5_, WD_2.5_, R_10_, CD_10_, or WD_10_ at 37ºC, 5% CO_2_ in a humidified environment for 20 h. Following exposure to PM, PBMC were infected with *M*.*tb* at MOI1 (**A, B, C, D**) or stimulated with PPD (**E, F, G, H**) and incubated for an additional 20 h. Frequencies of IFN-γ-producing PBMC were examined by ELISPOT assays. Results are shown as means ± SEM. Statistical significance (*p*≤0.05) relative to unexposed control PBMC is shown with solid lines. Statistically significant differences between seasons are shown with dotted lines.

In IGRA- PBMC, *M*.*tb*-induced IFN-γ spot frequencies were reduced upon exposure to R_2.5_, CD_10_ and WD_10_ at the 10 μg/ml dose only (Fig 4A and 4B). In IGRA+ subjects, however, frequencies of *M*.*tb*-induced IFN-γ-producing PBMC were suppressed in PBMC pre-exposed to 10 μg/ml PM_2.5_ and PM_10_ from all seasons (Fig 4C and 4D). The robust suppression of the frequencies of *M*.*tb*-induced IFN-γ-producing PBMC (>85%) relative to unexposed *M*.*tb*-infected PBMC observed upon pre-exposure to CD_10_ and WD_10_ (10 μg/ml, Fig 4D), may in part be due to PM-induced loss of cell viability (Fig 1D). Similarly, strong suppression of the frequencies of PPD-induced IFN-γ-producing cells was observed in PBMC from IGRA+ subjects when exposed to PM_2.5_ or PM_10_ from all seasons (Fig 4G and 4H). On the contrary, suppression of PPD-induced IFN-γ production in PBMC from IGRA- subjects was observed only upon pre-exposure to CD_2.5_, CD_10_ and WD_10_ (Fig 4E and 4F). As expected, the frequencies of *M*.*tb*- and PPD-induced IFN-γ producing PBMC were higher in PBMC from IGRA+ subjects (Fig 4C, 4D, 4G and 4H) than IGRA- subjects (Fig 4A, 4B, 4E and 4F) due to the higher frequency of *M*.*tb*-specific memory T cells, the primary producers of IFN-γ, in PBMC from IGRA+ subjects.

PM season-specific effects on frequencies of IFN-γ-producing cells were observed in *M*.*tb*-infected PBMC following pre-exposure to CD_10_ compared to R_10_ at 1 and 10 μg/ml in IGRA+ subjects (Fig 4D) and WD_10_ compared to R_10_ at 0.1, 1 and 10 μg/ml in both IGRA- and IGRA+ subjects (Fig 4B–4D). Furthermore, PM size-specific effects on frequencies of IFN-γ-producing cells were observed in *M*.*tb*-infected PBMC comparing PM_10_ and PM_2.5_ from the R and CD seasons at 10 μg/ml and the WD season at 0.1, 1 and 10 μg/ml, irrespective of the IGRA status (Table 2). Although the differences between PM exposures from different seasons were small, nevertheless they were statistically significant in PBMC pre-exposed to high concentrations of PM followed by *M*.*tb* infection.

**Table 2:** Suppressive effect of PM size on *M*.*tb*-induced IFN-γ production.

	R_10_ vs R_2.5_			CD_10_ vs CD_2.5_			WD_10_ vs WD_2.5_		
	0.1 μg/ml	1 μg/ml	10 μg/ml	0.1 μg/ml	1 μg/ml	10 μg/ml	0.1 μg/ml	1 μg/ml	10 μg/ml
**IGRA-**	n.s	n.s	**R**_**10**_**>R**_**2.5**_, **p = 0.004**	n.s	n.s	**CD**_**10**_**>CD**_**2.5**_**, *p* = 0.021**	**WD**_**10**_**>WD**_**2.5**_**, *p* = 0.029**	**WD**_**10**_**>WD**_**2.5**_**, *p* = 0.055**	**WD**_**10**_**>WD**_**2.5**_**, *p* = 0.030**
**IGRA+**	n.s	n.s	**R**_**10**_**>R**_**2.5**_**, *p* = 0.048**	n.s	n.s	**CD**_**10**_**>CD**_**2.5**_**, *p* = 0.0028**	**WD**_**10**_**>WD**_**2.5**_**, *p* = 0.028**	**WD**_**10**_**>WD**_**2.5**_**, *p* = 0.031**	**WD**_**10**_**>WD**_**2.5**_**, *p* = 0.012**

n.s. not statistically significant

**Fig 5:**
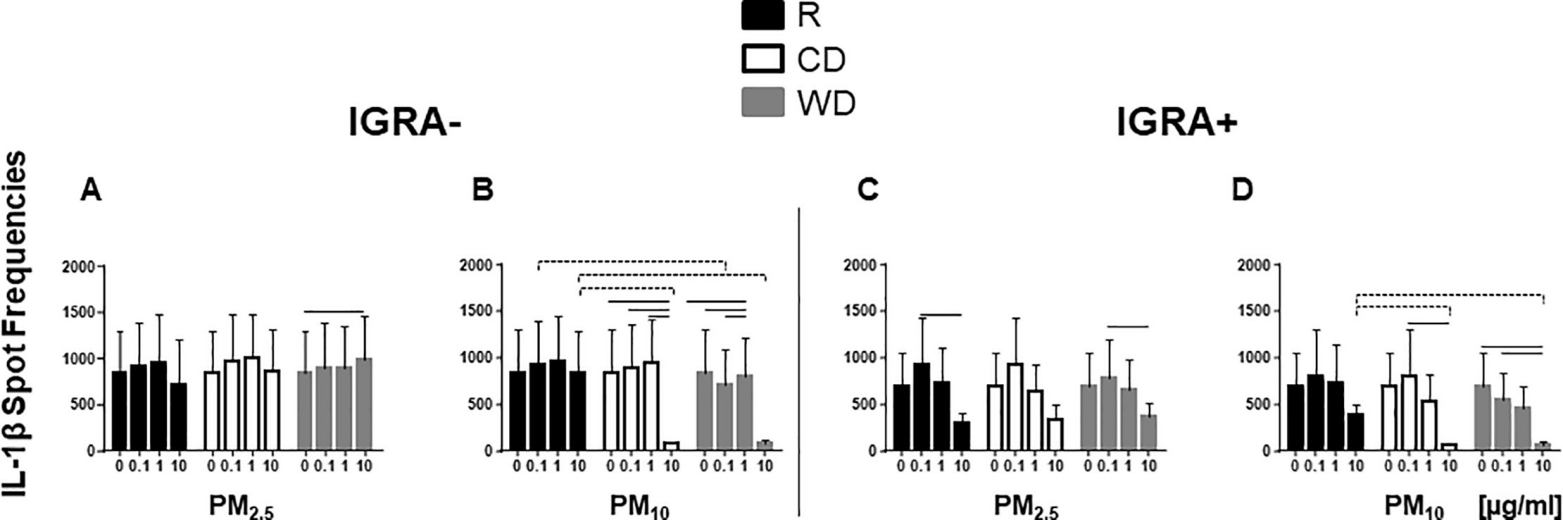
Effect of PM on *M*.*tb*-induced IL-1β production. PBMC from IGRA- (n = 7) or IGRA+ (n = 7) subjects were exposed to 0 (no PM), 0.1, 1 and 10 μg/ml of R_2.5_, CD_2.5_, WD_2.5_, R_10_, CD_10_, or WD_10_ at 37ºC, 5% CO2 in a humidified environment for 20 h. Following Exposure to PM, PBMC were infected with *M*.*tb* at MOI1 or left uninfected (**A, B, C,** and **D**) and incubated for additional 20 h. Frequencies of IL-1β-producing PBMC were examined by ELISPOT assays (see Materials and Methods). Results are shown as means ± SEM. Statistically significant (*p*≤0.05) dose-dependent changes relative to unexposed control PBMC is shown with solid lines. Statistically significant differences between seasons are shown with dotted lines.

In contrast to the higher frequencies of *M*.*tb*-induced IFN-γ-producing PBMC in IGRA- subjects, no differences in frequencies of IL-1β-producing PBMC were observed between IGRA- and IGRA+ subjects (Fig 5A–5D). Pre-exposure to CD_10_ and WD_10_ suppressed the frequencies of IL-1β-producing cells at 10 μg/ml concentrations irrespective of the IGRA status of the study subjects (Fig 5B–5D). Unlike our findings with IFN-γ production, which showed that PM_10_ within each season was more suppressive than PM_2.5_ (Table 2), no statistically significant effects of PM size were observed in any of the three seasons on frequencies of *M*.*tb*-induced IL-1β-producing PBMC.

### PM size and season effects on *M*.*tb* growth control

**Fig 6:**
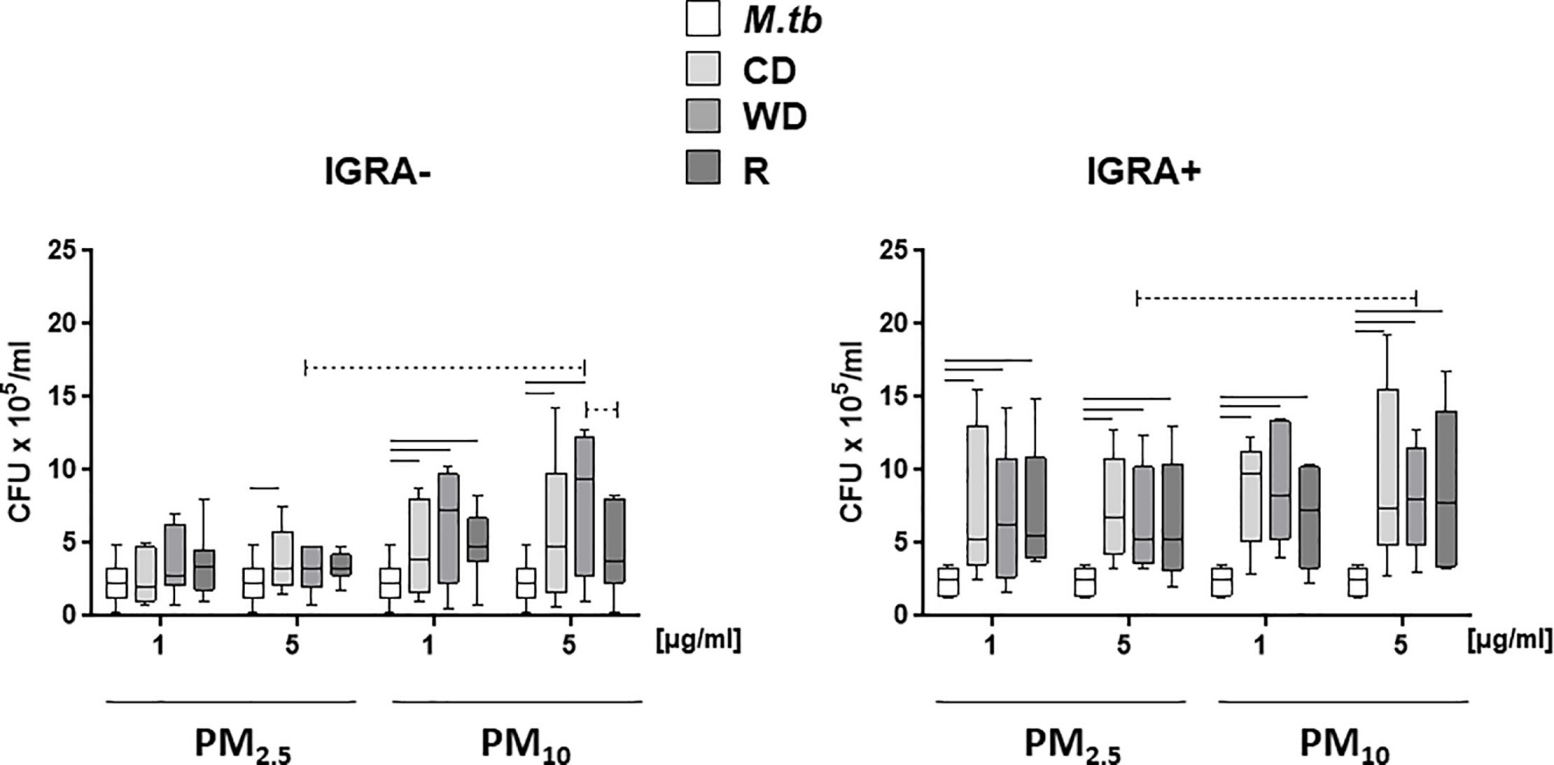
Effect of PM size and seasonal source on *M*.*tb* growth control. PBMC from IGRA- (n = 7) or IGRA+ (n = 5) subjects were exposed to 0 (no PM), 1 and 5 μg/ml of R_2.5_, CD_2.5_, WD_2.5_, R_10_, CD_10_, or WD_10_ at 37ºC, 5% CO2 in a humidified environment for 20 h. Following pre-exposure to PM, PBMC were infected with *M*.*tb* at MOI1 or left uninfected. After 2 h infection, non-phagocytosed *M*.*tb* was removed by washing and plates subsequently incubated in complete culture media. PBMC were lysed and serial dilutions of cell lysates plated in *M*.*tb* growth media in triplicate on 7H10 agar plates and incubated at 37°C for 21 days until *M*.*tb* colony forming units (cfu) were determined. Results from day 4 are shown. Statistically significant (*p*≤0.05) differences relative to PM-unexposed *M*.*tb*-infected control PBMC are shown with solid lines. Size and season-specific differences are shown with dotted lines.

To examine whether the observed PM-mediated suppression of *M*.*tb*-induced IFN-γ and IL-β-production by PBMC is associated with alterations in the control of *M*.*tb* growth in PBMC, we exposed PBMC from IGRA- and IGRA+ subjects to PM_2.5_ and PM_10_ (0, 1 and 5 μg/ml) from the three seasons followed by infection with *M*.*tb* at MOI 1. Pre-exposure of PBMC to PM_10_ (1 and 5 μg/ml) suppressed the control of *M*.*tb* growth (i.e. increased cfu numbers) by PBMC from both IGRA- and IGRA+ subjects to a greater extent than pre-exposure to PM_2.5_ on days 0, 1 and 4 (Table 3). Results from day 4 are shown in Fig 6. On day 4, pre-exposure to WD_10_ at 5 μg/ml resulted in a greater suppression of *M*.*tb* growth control than WD_2.5_ at 5 μg/ml in both IGRA- and IGRA+ PBMC (Table 3, Fig 6). On day 1, pre-exposure to R_10_ induced greater suppression of *M*.*tb* growth control than R_2.5_ in both IGRA- and IGRA+ PBMC (Table 3). Season-specific differences between WD_10_ and R_10_ were observed in IGRA- subjects (Fig 6). *M*.*tb* growth control on day 7 could not be interpreted because no viable PBMC were left due to cell death from PM-induced cytotoxicity (data not shown). Statistical significances (*p*-values) of PM size-specific differences between WD_10_ and WD_2.5_ and R_10_ and R_2.5_ are shown in Table 3. For easier understanding, comparisons of cytotoxicity and host responses upon seasonal PM exposure followed by *M*.*tb* infection or PPD stimulation are shown in Table 4.

**Table 3:** PM size effect on *M*.*tb* growth control.

IGRA Status	Dose [μg/ml]	Day	PM_10_ vs PM_2.5_	*p*-value
-	5	4	WD_10_˃WD_2.5_	**0.032**
+	5	4	WD_10_˃WD_2.5_	**0.02**
-	1	0	R_10_>R_2.5_	**0.0095**
+	1	0	R_10_ vs R_2.5_ (n.s.)	0.51
-	1	1	R_10_>R_2.5_	**0.0029**
+	1	1	R_10_>R_2.5_	**0.016**
-	5	1	R_10_>R_2.5_	**0.026**
+	5	1	R_10_ vs R_2.5_ (n.s.)	0.32

n.s. not statistically significant

**Table 4:** PM-induced cytotoxicity and *M*.*tb*-induced host immune responses in seasonal PM-pre-exposed PBMC.

Increased Host Response	R_2.5_	CD_2.5_	WD_2.5_	R_10_	CD_10_	WD_10_
PM-induced Cytotoxicity (LDH, Fig 1A–1D)	n.s.	n.s.	n.s.	n.s.	+	+
Phagocytosis (% *M*.*tb* uptake, Fig 2)	-	-	n.s.	-	-	-
PM-induced IFN-γ (spot frequency (Fig 3A–3D)	+	+	+	+	+	+
PM-induced IL-1β (spot frequency (Fig 3E–3H)	+	+	n.s.	+	n.s.	n.s.
PM + M.tb-induced IFN-γ in IGRA-/IGRA+ subjects (spot frequency, Fig 4A–4D)	-/-	n.s./-	n.s./-	n.s./-	-/-	-/-
PM + PPD-induced IFN-γ in IGRA-/IGRA+ subjects (spot frequency, Fig 4E–4H)	n.s./-	n.s./-	n.s./-	n.s./-	-	-
PM + *M*.*tb*-induced IL-1β in IGRA-/IGRA+ subjects (spot frequency, Fig 6A–6D)	n.s./-	n.s./n.s.	+/-	n.s./n.s.	-/-	-/-
Loss of *M*.*tb* growth control (CFU on day 4, 5μg/ml, Fig 7)	n.s./+	+/+	n.s./+	n.s./+	+/+	+/+

n.s. indicates no statistical significance relative to control unexposed PBMC; + or–indicates statistically significant increase (+) or decrease (-) relative to control PBMC; / indicates comparison between IGRA- and IGRA+ subjects.

### PM exposure and its effect on *M*.*tb*-induced signaling pathways

Finally, we examined whether the observed PM-mediated suppression of *M*.*tb*-induced IFN-γ expression could result from a PM-mediated modulation of T-bet, a T-box transcription factor involved in the regulation of IFN-γ production by T cells. Pre-exposures to R_2.5_, CD_2.5_, and WD_2.5_ did not cause cytotoxicity to PBMC (Fig 1A–1C) even at the highest concentrations despite of the capacity of the PM to suppress *M*.*tb*-induced IFN-γ-production by PBMC relative to unexposed *M*.*tb*-infected PBMC (Fig 4A–4C). In contrast to PM_2.5_, pre-exposure to PM_10_ caused cytotoxicity at the 10 μg/ml concentration.

**Fig 7:**
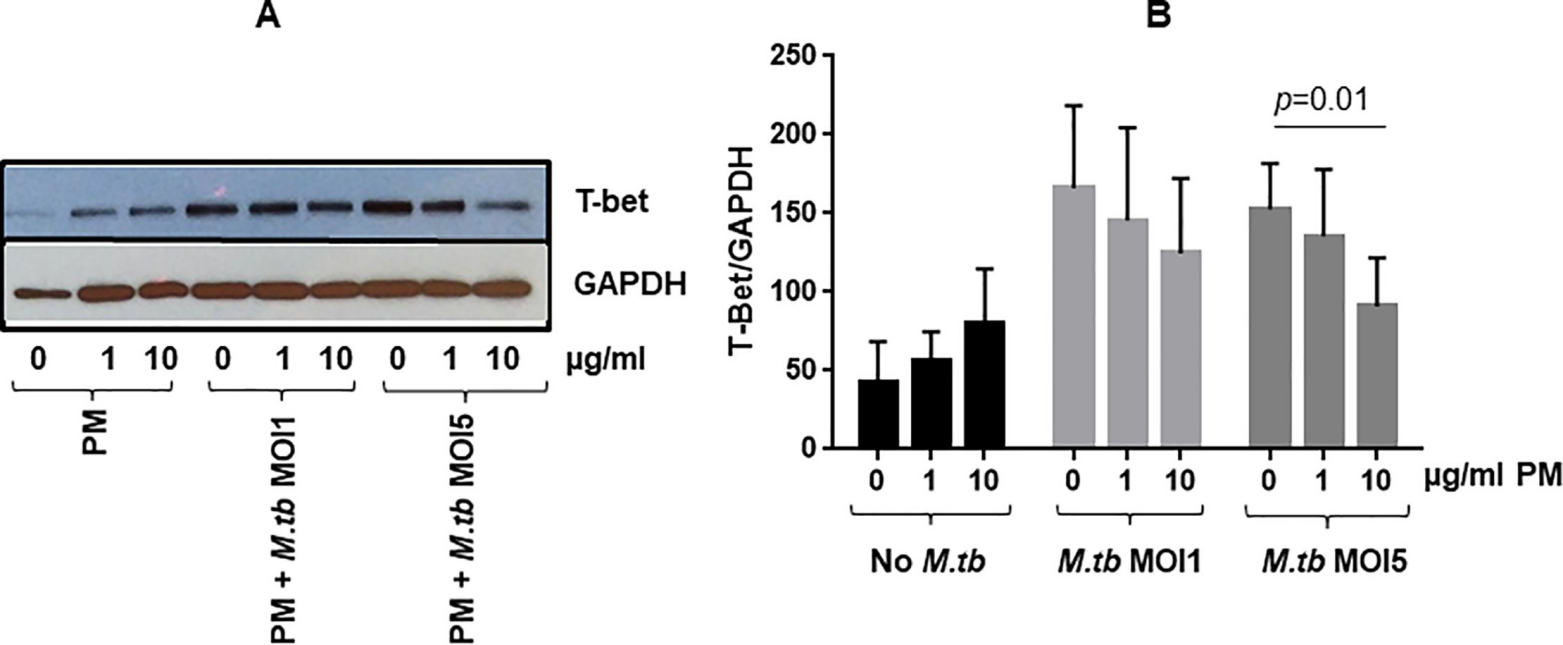
Effect of PM on T-bet expression in PBMC. PBMC (n = 3) were pre-exposed to 0, 1 and 10 μl/ml PM_2.5_ for 24 h followed by infection with *M*.*tb* at MOI1 and 5 or left uninfected for 24h. Protein lysates (see Materials and Methods) were analyzed with SDS/PAGE followed by western blotting with T-bet and GAPDH antibodies. (A) T-Bet and GAPDH expression in one representative of three independent experiments from three subjects (S1 Fig); (B) T-Bet and GAPDH specific bands were quantitated by ImageJ and the ratio of percentage of T-bet and GAPDH (normalized) for each condition were plotted as a function of PM concentration (Mean ± SEM from three independent experiments involving three different subjects). Statistical significances (*p*≤0.05) of PM-exposed relative to unexposed PBMC that were uninfected or infected with *M*.*tb* were analyzed by student t test (2-tailed pairwise comparison) and *p*-value is shown.

It was therefore decided to use PM_2.5_ only for the following experiments. PBMC were exposed to PM_2.5_ for 24 h and infected with *M*.*tb* at MOI1 and 5, or left uninfected. As in our earlier study [21]_,_ for this set of experiments, annual bulk PM_2.5_ was used and *M*.*tb* infections were performed at MOI 1 and 5. T-bet and GAPDH expression from a representative experiment is shown in Fig 7A. As evidenced from the normalized data, T-bet expression increased in PM_2.5_-exposed PBMC as well as upon *M*.*tb* infection at MOI1 and MOI5. Interestingly, in contrast, *M*.*tb*-induced T-bet levels were reduced in PM_2.5_ pre-exposed and *M*.*tb* (MOI5)-infected PBMC compared to *M*.*tb* (MOI5)-infected PBMC (Fig 7B). These findings suggest that PM_2.5_-mediated reduction in T-bet expression contributes to the reduction of *M*.*tb*-induced IFN-γ expression in PM-pre-exposed PBMC.

## Discussion

*M*.*tb* infection is endemic in many, primarily, low-income countries of the world where air pollution is on the rise due to growing urbanization. Seasonal variations of TB incidence rates with highest rates being in springs and summers have been observed in some studies globally [35, 44–46]. Alterations in human immune responses, co-infections with seasonal respiratory viruses, increased indoor exposures during winter months and seasonal differences in humidity or temperature [44] have been attributed to TB seasonality. However, the roles of urban air pollution exposures and seasonal variations in PM composition in determining seasonality of TB are inconclusive. To address these knowledge gaps, effects of exposures to urban PM_2.5_ and PM_10_ from different seasons on *M*.*tb*-induced inflammatory responses, *M*.*tb* phagocytosis and *M*.*tb* growth control in PBMC were examined in the current study.

PM exposure of PBMC prior to *M*.*tb* infection suppressed *M*.*tb* phagocytosis. This may be explained by a competitive engagement of PM with cellular *M*.*tb* uptake mechanisms, for example via scavenger receptors and/or TLR interactions [47]. Seasonal differences in proportions of organic components in PM may modify such mechanisms.

To examine whether PM may alter critical *M*.*tb*-specific protective human host immune responses, we focused on the *M*.*tb*-induced IL-1β and IFN-γ production by PBMC. IL-1β production by monocytes/macrophages is pivotal for intracellular *M*.*tb* growth control in macrophages [48]. IFN-γ activates macrophages to confer immunity to *M*.*tb* and is released primarily from CD4^+^ and CD8^+^ T-cells, which upon stimulation can expand in the respiratory tract or extravasate from the systemic circulation into the lung compartment. Urban air pollution PM impaired IFN-γ responses following *M*.*tb* infection (Fig 4A–4D) or stimulation with PPD (Fig 4E–4H), reminiscent of our earlier studies with diesel exhaust particles [19].

Inclusion of IGRA+ and IGRA-subjects in the current study, allowed us to examine PM effects on *M*.*tb*-induced PBMC responses in the context of immunological memory to prior *M*.*tb* infection. As expected, frequencies of *M*.*tb*-antigen-specific IFN-γ-producing PBMC were higher in IGRA+ than IGRA- subjects (Fig 4C, 4D, 4G and 4H). In contrast, to *M*.*tb*-induced IFN-γ, the *M*.*tb*-induced IL-1β production was independent of the IGRA status of the study subjects as IL-1β is a product of innate immune cells and T cell memory-independent.

Seasonal changes in anthropogenic and soil elements in dry and wet seasons in Mexico City have been reported [39]. Further, seasonal variation of PAH content in PM_10_ in Mexico City has been shown to correlate with differences in toxicity [49]. In the current study, CD_10_ and WD_10_-mediated suppression of frequencies of *M*.*tb*-induced IFN-γ and IL-1β-producing PBMC (Figs 5B, 5D, 6B and 6D) may in part be due to the cytotoxic effects of the PM at 10 μg/ml concentration. CD_10_ or WD_10,_ induced more cytotoxicity in PBMC than the other four PM samples (Fig 1B and 1D). R_10_ had a significantly higher capacity to stimulate the production of IFN-γ than CD_10_ or WD_10_ (Fig 3B–3D). Taken together, seasonal changes in the chemical composition of PM from rainy and dry seasons may account for observed differences in biological responses.

To examine whether the modulation of the cytokine profiles of PBMC following PM pre-exposure affects the growth control of *M*.*tb* by PBMC, we compared exposure effects of seasonal PM in PBMC from IGRA+ and IGRA- subjects. Both PM_2.5_ and PM_10_ induced loss of *M*.*tb* growth control in IGRA+ subjects, whereas only PM_10_ induced loss of *M*.*tb* growth control on IGRA- subjects (Fig 6). Further, PBMC pre-exposure to WD_10_ (Table 2) induced a significantly greater loss of *M*.*tb* growth control than WD_2.5_ (Fig 6), which is consistent with the greater IFN-γ suppression effect noted upon WD_10_ exposure (Table 2).Significant differences in the chemical composition (S1 Table) of PM_2.5_ and PM_10_, may be important drivers of the inhibitory effects on *M*.*tb* growth control (Fig 6).

To gain insights into the mechanisms underlying the observed PM-mediated suppression of the frequencies of IFN-γ-producing PBMC, we examined if PM_2.5_ affects the expression of T-bet, a transcription factor known to regulate IFN-γ expression in T cells. Interestingly, PM_2.5_ exposure prior to *M*.*tb* infection led to a reduction of T-bet expression, particularly in *M*.*tb* MOI5-infected PBMC (Fig 7), thus potentially contributing to the suppression of IFN-γ-production by PBMC.

The analysis of PM composition showed significant differences in organic and inorganic components between PM_2.5_ and PM_10_ (S1 Table). Differences in the chemical composition in PM_2.5_ and PM_10_ between the seasons were less evident (S2 and S3 Tables). The different functional responses observed here cannot be linked with certainty to differences in specific chemical components of the PM alone, as interactions among chemical components of the PM may be additional confounders [27].

Overall, the current study shows that differences in the biological responses of PBMC to PM in some instances were related to the presence of PM only, and in others to PM size or season or a combination of all. Future studies may have to specifically focus on the biological effects of chemical components of air pollution PM_10_ and PM_2.5_ and their emission sources as such studies may help identify specific high impact targets for regulatory interventions to protect public health.

The findings from this study suggest that complex interactions between cell surface receptors and PM and its chemical components underlie air pollution exposure effects on *M*.*tb* host immune responses. In addition, the effects of real-world air pollution exposures and their biological consequences are likely far more complex than what can be modelled in experiments, such as those chosen in the current study. Air pollution effects are mediated by complex mixtures of gases and volatile compounds in addition to PM, and immune cell exposures in the respiratory spaces occur in the context of interactions with respiratory epithelial cells and a multitude of bioactive proteins including surfactants found at the air-fluid-cell interface. However, despite these limitations, our study demonstrates that concentration and size of PM and seasonal differences in PM composition differentially impair *M*.*tb*-induced cytokine production and *M*.*tb* growth control and thus modify crucial components of human antimycobacterial host immune responses. These findings may have public health implications that go beyond the risk of increased rates of TB development in air polluted environments. Given the observed alterations of *M*.*tb* and PPD-induced cytokine responses following PM exposures, one may speculate that the performance of *M*.*tb* antigen-based immunodiagnostic (IGRA assays) and immune responses to novel anti-tuberculous vaccines may be modified in air-polluted environments.

In summary, the findings from this study provide biological plausibility supporting the growing epidemiological evidence that exposure to urban air pollution PM influences the outcome (e.g., seasonality) of *M*.*tb* infections and TB treatment. Air quality, PM content and PM composition in different seasons likely modify the natural course of *M*.*tb* infection (incidence rates, disease outcomes) differentially in different areas of the world.

## Supporting information

S1 Table Mean concentrations (ng/mg) of chemical components that are significantly different between PM_10_ and PM_2.5_. (DOCX)

S2 Table Differences in chemical component concentrations (ng/ml) in seasonal PM_10_. (DOCX)

S3 Table Differences in chemical component concentrations (ng/ml) in seasonal PM_2.5_. (DOCX)

S4 Table Endotoxin levels in monthly PM samples used for the study. (DOCX)

S1 Fig T-bet and GAPDH expression in three subjects. (PPTX)

S1 Data Raw data for Fig 1. (XLS)

S2 Data Raw data for Fig 2. (XLSX)

S3 Data Raw data for Fig 3. (XLS)

S4 Data Raw data for Fig 4. (XLS)

S5 Data Raw data for Fig 5. (XLS)

S6 Data Raw data for Fig 6. (XLSX)

S7 Data Raw data for Fig 7. (XLSX)
