# Supplementary material for: Season and size of urban particulate matter differentially affect cytotoxicity and human immune responses to Mycobacterium tuberculosis
Source: PLoS One. 2019 Jul 11;14(7):e0219122. doi: 10.1371/journal.pone.0219122 (PMC6622489; doi:10.1371/journal.pone.0219122)
Supplement: S1 Table — (DOCX) [file pone.0219122.s001.docx]

**S1 Table.** Mean concentrations (ng/mg) of chemical components that are significantly different between PM_10_ and PM_2.5_.

|  |  | **PM_10_ (N=12)** | | | | **PM_2.5_ (N=12)** | | | |
| --- | --- | --- | --- | --- | --- | --- | --- | --- | --- |
| **Variable** | **Label** | **Mean** | **Std. Dev.** | **Min.** | **Max.** | **Mean** | **Std. Dev.** | **Min.** | **Max.** |
| A54C | n-tricosane (n-C23) | 52.5 | 15.2 | 32.7 | 86.2 | 28.0 | 20.0 | 0.0 | 59.5 |
| A61C | n-triacontane (n-C30) | 83.6 | 49.8 | 0.0 | 191.7 | 189.1 | 72.9 | 90.9 | 310.7 |
| H92C | abS-homohopane (C31abS-hopane) | 10.4 | 2.6 | 5.8 | 14.9 | 7.1 | 1.9 | 3.2 | 10.3 |
| H97C | 22S-trishomohopane (C33) | 6.3 | 1.7 | 3.1 | 8.6 | 3.5 | 1.3 | 1.8 | 6.4 |
| A53C | n-docosane (n-C22) | 32.5 | 12.8 | 0.0 | 49.1 | 15.4 | 14.5 | 0.0 | 36.6 |
| A64C | n-tritriactotane (n-C33) | 114.1 | 48.2 | 53.0 | 224.9 | 206.5 | 77.0 | 104.9 | 315.1 |
| H87C | 22,29,30-norhopane (29Ts) | 4.5 | 1.0 | 2.9 | 5.9 | 2.1 | 1.9 | 0.0 | 4.8 |
| H93C | abR-homohopane (C31abR-hopane) | 26.2 | 7.4 | 10.4 | 38.6 | 17.9 | 4.4 | 10.4 | 25.3 |
| A59C | n-octacosane (n-C28) | 113.9 | 47.5 | 61.4 | 229.3 | 207.5 | 86.6 | 102.2 | 352.8 |
| A63C | n-dotriacontane (n-C32) | 69.1 | 36.2 | 0.0 | 142.2 | 155.2 | 58.0 | 69.8 | 246.2 |
| A55C | n-tetracosane (n-C24) | 67.6 | 27.9 | 36.7 | 140.0 | 44.5 | 28.8 | 0.0 | 98.6 |
| A62C | n-hentriacotane (n-C31) | 186.3 | 78.6 | 70.3 | 367.6 | 314.3 | 123.7 | 154.2 | 489.1 |
| H89C | ab-hopane (C30ab -hopane) | 25.3 | 6.3 | 14.3 | 34.7 | 17.5 | 4.4 | 10.5 | 25.0 |
| H86C | ab-norhopane (C29ab-hopane) | 26.9 | 6.9 | 14.3 | 38.9 | 18.6 | 4.9 | 10.5 | 27.3 |
| H98C | 22R-trishomohopane (C33) | 3.4 | 2.0 | 0.0 | 6.8 | 1.0 | 1.0 | 0.0 | 2.8 |
| S104C | abb 20R-Cholestane | 3.3 | 2.0 | 0.0 | 6.4 | 0.9 | 1.1 | 0.0 | 3.0 |
| A67C | n-hexatriacontane (n-C36) | 88.9 | 37.5 | 0.0 | 150.7 | 164.9 | 50.7 | 82.6 | 228.2 |
| H96C | abR-bishomohopane (C32abR-hopane) | 6.3 | 2.3 | 1.4 | 8.6 | 3.4 | 1.9 | 0.0 | 7.3 |
| P23C | indeno[1,2,3-cd]pyrene | 2.2 | 3.5 | 0.0 | 9.0 | 9.3 | 7.1 | 0.0 | 21.2 |
| A65C | n-tetratriactoane (n-C34) | 56.0 | 29.5 | 0.0 | 116.0 | 117.6 | 58.9 | 0.0 | 192.4 |
| H88C | aa- + ba-norhopane (C29aa- + ba -hopane) | 2.3 | 1.4 | 0.0 | 4.3 | 0.8 | 1.0 | 0.0 | 2.3 |
| A60C | n-nonacosane (n-C29) | 153.1 | 80.3 | 54.5 | 345.3 | 259.0 | 135.2 | 61.4 | 481.6 |
| A58C | n-heptacosane (n-C27) | 165.3 | 74.5 | 91.1 | 348.2 | 231.7 | 109.6 | 100.3 | 423.2 |
| H84C | 22,29,30-trisnorneophopane (Ts) | 5.6 | 3.0 | 0.0 | 9.5 | 2.4 | 2.5 | 0.0 | 5.2 |
| A66C | n-pentatriacontane (n-C35) | 79.6 | 39.4 | 0.0 | 148.2 | 144.0 | 72.3 | 0.0 | 228.3 |
| H91C | Ba-hopane (C30ba –hopane) | 2.4 | 1.7 | 0.0 | 5.4 | 0.7 | 1.1 | 0.0 | 3.2 |
| S112C | Abb 20R 24R-Ethylcholestane | 2.5 | 3.0 | 0.0 | 5.4 | 0.3 | 0.9 | 0.0 | 3.1 |
| S114C | Aaa 20R 24R-Ethylcholestane | 2.3 | 2.5 | 0.0 | 6.0 | 0.7 | 1.6 | 0.0 | 4.8 |
| N4CC | Ammonium | 7494.2 | 2057.1 | 4297.1 | 11269.1 | 19065.9 | 5607.7 | 5437.0 | 26816.1 |
| CAAC | Soluble Calcium | 23596.8 | 3640.9 | 18745.8 | 29165.9 | 13971.6 | 6182.4 | 8382.5 | 30546.1 |
| SUXC | Sulfur | 26531.1 | 6532.5 | 17453.6 | 34901.7 | 39438.2 | 10163.9 | 19146.7 | 57869.2 |
| N3IC | Nitrate | 14155.6 | 5982.7 | 6678.7 | 27117.2 | 9141.4 | 3906.3 | 4302.3 | 16216.3 |
| CAXC | Calcium | 32745.3 | 5109.8 | 22903.1 | 38102.4 | 21160.4 | 9804.5 | 13363.6 | 45714.0 |
| MGAC | Soluble Magnesium | 1395.1 | 229.1 | 1016.0 | 1805.5 | 974.8 | 294.0 | 570.3 | 1553.3 |
| CLIC | Chloride | 1675.8 | 1080.9 | 129.5 | 4019.1 | 621.1 | 383.2 | 160.3 | 1380.2 |
| SEXC | Selenium | 20.1 | 21.3 | 5.6 | 82.6 | 44.8 | 33.9 | 8.3 | 123.8 |
| O2TC | Organic Carbon Fraction 2 | 30900.4 | 8424.0 | 19093.8 | 47726.7 | 46071.8 | 19211.3 | 19389.0 | 74845.7 |
| S4IC | Sulfate | 63319.5 | 15600.5 | 38275.4 | 84325.6 | 81644.0 | 19330.5 | 32982.0 | 107069.1 |
| PBXC | Lead | 352.7 | 107.7 | 153.7 | 503.2 | 621.4 | 245.5 | 275.5 | 1257.1 |
| E3TC | Elemental Carbon Fraction 3 | 3633.2 | 1387.2 | 1841.8 | 6995.1 | 1895.8 | 1365.0 | 88.4 | 3954.1 |
| NAXC | Sodium qualitative only | 3645.8 | 7344.0 | 773.4 | 26773.4 | 16688.9 | 10870.7 | 1714.9 | 35623.7 |
| BRXC | Bromine | 103.3 | 32.5 | 51.3 | 161.5 | 176.4 | 78.3 | 80.7 | 355.6 |
| ZNXC | Zinc | 2251.8 | 857.2 | 1265.4 | 4124.4 | 3081.6 | 912.2 | 1761.7 | 4895.6 |
| NAAC | Soluble Sodium | 3524.6 | 525.8 | 2819.9 | 4544.7 | 3006.2 | 545.0 | 2086.1 | 4236.2 |
| CLXC | Chlorine | 2160.3 | 1223.1 | 571.1 | 4683.1 | 1279.3 | 705.7 | 395.5 | 2814.5 |
| VAXC | Vanadium | 284.1 | 141.2 | 104.0 | 533.6 | 479.8 | 339.9 | 91.1 | 1310.7 |
| PHXC | Phosphorous | 87.8 | 80.9 | 10.6 | 237.8 | 23.3 | 24.5 | 8.8 | 99.1 |

Min., minimum; max., maximum; Std. Dev., standard deviation
