# Supplementary material for: Season and size of urban particulate matter differentially affect cytotoxicity and human immune responses to Mycobacterium tuberculosis
Source: PLoS One. 2019 Jul 11;14(7):e0219122. doi: 10.1371/journal.pone.0219122 (PMC6622489; doi:10.1371/journal.pone.0219122)
Supplement: S2 Table — (DOCX) [file pone.0219122.s002.docx]

**S2 Table.** Differences in chemical component concentrations (ng/ml) in seasonal PM

| **Season** | **Variable** | **Label** | **Mean** | **Std Dev** | **Minimum** | **Maximum** |
| --- | --- | --- | --- | --- | --- | --- |
| R10 | H91C | ba-hopane (C30ba -hopane) | 2.6068087 | 0.7136299 | 1.6389135 | 3.5058145 |
|  | P20C | benzo[e]pyrene | 13.0791245 | 2.9512877 | 8.7279051 | 16.4326692 |
|  | P18C | benzo[j+k]fluoranthene | 53.1847711 | 12.8385122 | 32.9901698 | 66.6256477 |
|  | P32C | benzo(ghi)fluoranthene | 8.7904507 | 2.5995998 | 5.0715225 | 11.6638645 |
|  | P21C | benzo[a]pyrene | 5.3551054 | 1.7583765 | 4.0008551 | 8.2637433 |
| CD10 | H91C | ba-hopane (C30ba -hopane) | 3.9310241 | 0.9767540 | 3.2212510 | 5.3558959 |
|  | P20C | benzo[e]pyrene | 26.5676065 | 5.9571495 | 21.8363916 | 34.5102355 |
|  | P18C | benzo[j+k]fluoranthene | 104.2382619 | 12.4752728 | 93.1298416 | 121.9410545 |
|  | P32C | benzo(ghi)fluoranthene | 13.0335558 | 1.8948383 | 10.7807426 | 15.4139780 |
|  | P21C | benzo[a]pyrene | 17.6374036 | 7.9110800 | 8.9622378 | 25.8505864 |
| WD10 | H91C | ba-hopane (C30ba -hopane) | 0 | 0 | 0 | 0 |
|  | P20C | benzo[e]pyrene | 3.0620077 | 5.3035529 | 0 | 9.1860231 |
|  | P18C | benzo[j+k]fluoranthene | 18.1161692 | 31.3781255 | 0 | 54.3485077 |
|  | P32C | benzo(ghi)fluoranthene | 1.9200046 | 3.3255455 | 0 | 5.7600138 |
|  | P21C | benzo[a]pyrene | 0 | 0 | 0 | 0 |

Particles with significantly (*p*< 0.05) larger mean concentrations (ng/mg) within PM_10_ are highlighted in grey.
