# Supplementary material for: Season and size of urban particulate matter differentially affect cytotoxicity and human immune responses to Mycobacterium tuberculosis
Source: PLoS One. 2019 Jul 11;14(7):e0219122. doi: 10.1371/journal.pone.0219122 (PMC6622489; doi:10.1371/journal.pone.0219122)
Supplement: S3 Table — (DOCX) [file pone.0219122.s003.docx]

**S3 Table.** Seasonal Particles with highest levels of components in PM_2.5_ are highlighted in grey.

|  | **Variable** | **Label** | **Mean** | **Std Dev** | **Minimum** | **Maximum** |
| --- | --- | --- | --- | --- | --- | --- |
| R_2.5_ | H91C | ba-hopane (C30ba -hopane) | 0.8392374 | 1.3752196 | 0 | 3.1663913 |
|  | P20C | benzo[e]pyrene | 11.2120471 | 8.1515357 | 0 | 21.7893894 |
| CD_2.5_ | H91C | ba-hopane (C30ba -hopane) | 0.5284633 | 1.0569267 | 0 | 2.1138534 |
|  | P20C | benzo[e]pyrene | 31.5124112 | 9.5856481 | 17.7805717 | 39.9082500 |
| WD_2.5_ | H91C | ba-hopane (C30ba -hopane) | 0.7083332 | 1.2268690 | 0 | 2.1249995 |
|  | P20C | benzo[e]pyrene | 11.0880574 | 9.6454063 | 0 | 17.5404365 |
