# Supplementary material for: Season and size of urban particulate matter differentially affect cytotoxicity and human immune responses to Mycobacterium tuberculosis
Source: PLoS One. 2019 Jul 11;14(7):e0219122. doi: 10.1371/journal.pone.0219122 (PMC6622489; doi:10.1371/journal.pone.0219122)
Supplement: S4 Table — (DOCX) [file pone.0219122.s004.docx]

**S4 Table.** Endotoxin levels in monthly PM samples used for the study

| **Year** | **Month** | **Endotoxin concentration (EU/mg dust)** | | **PM** |
| --- | --- | --- | --- | --- |
|  |  | **Average** | **Standard deviation** |  |
| 2012 | June | 2.348 | 0.278 |  |
| 2012 | July | <0.013 | n/a |  |
| 2012 | August | <0.043 | n/a | Rainy (R_2.5_) |
| 2012 | September | <0.012 | n/a |  |
| 2012 | October | 0.011 | 0.001 |  |
| 2012 | November | <0.012 | 0 |  |
| 2012 | December | <0.008 | 0 | Cold-dry (CD_2.5_) |
| 2013 | January | 0.005 | 0.001 |  |
| 2013 | February | 0.035 | 0 |  |
| 2013 | March | <0.008 | n/a |  |
| 2013 | April | 0.014 | 0.001 | Warm-dry (WD_2.5_) |
| 2013 | May | 0.008 | 0.001 |  |
| 2012 | June | <0.005 | n/a |  |
| 2012 | July | <0.003 | 0 |  |
| 2012 | August | <0.005 | n/a | Rainy (R_10_) |
| 2012 | September | 0.007 | 0 |  |
| 2012 | October | 0.018 | 0.002 |  |
| 2012 | November | <0.005 | n/a |  |
| 2012 | December | 0.037 | 0.002 | Cold-dry (CD_10_) |
| 2013 | January | 0.017 | 0.002 |  |
| 2013 | February | 0.076 | 0.004 |  |
| 2013 | March | 0.195 | 0.014 |  |
| 2013 | April | 0.044 | 0.005 | Warm-dry (WD_10_) |
| 2013 | May | 0.007 | 0 |  |

Endotoxin levels were determined by LAL assay. (Chromo-LAL assay, Cat. # C0031-25, Associates of Cape Cod, Inc., East Falmouth, MA).
