# Supplementary material for: Season and size of urban particulate matter differentially affect cytotoxicity and human immune responses to Mycobacterium tuberculosis
Source: PLoS One. 2019 Jul 11;14(7):e0219122. doi: 10.1371/journal.pone.0219122 (PMC6622489; doi:10.1371/journal.pone.0219122)
Supplement: S1 Fig — (PPTX) [file pone.0219122.s005.pptx]

## Slide 1
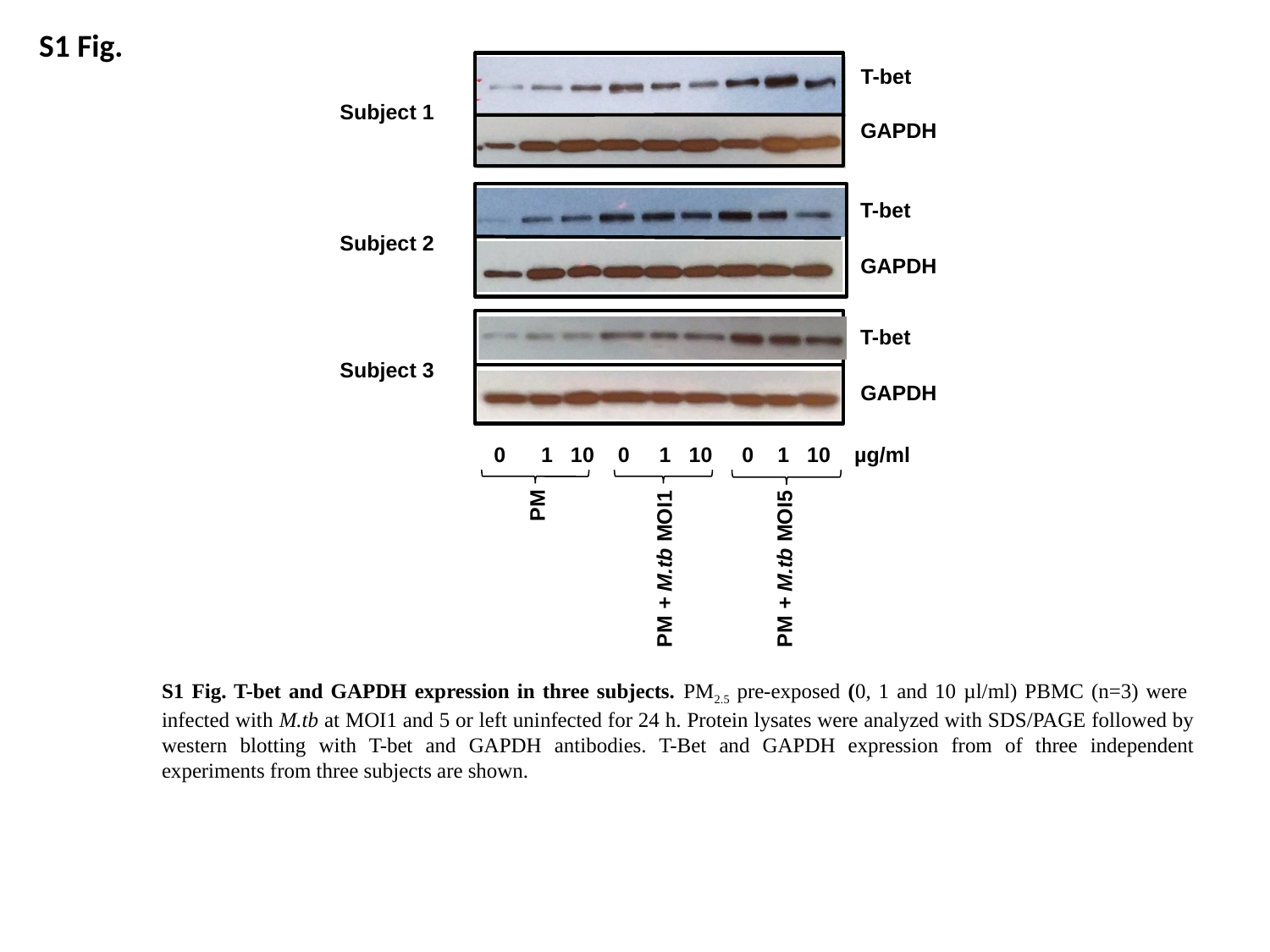

S1 Fig.
T-bet
Subject 1
GAPDH
T-bet
Subject 2
GAPDH
T-bet
Subject 3
GAPDH
0 1 10 0 1 10 0 1 10
µg/ml
PM
PM + M.tb MOI1
PM + M.tb MOI5
S1 Fig. T-bet and GAPDH expression in three subjects. PM2.5 pre-exposed (0, 1 and 10 µl/ml) PBMC (n=3) were infected with M.tb at MOI1 and 5 or left uninfected for 24 h. Protein lysates were analyzed with SDS/PAGE followed by western blotting with T-bet and GAPDH antibodies. T-Bet and GAPDH expression from of three independent experiments from three subjects are shown.
